# Improving Glyphosate Oxidation Activity of Glycine Oxidase from *Bacillus cereus* by Directed Evolution

**DOI:** 10.1371/journal.pone.0079175

**Published:** 2013-11-05

**Authors:** Tao Zhan, Kai Zhang, Yangyan Chen, Yongjun Lin, Gaobing Wu, Lili Zhang, Pei Yao, Zongze Shao, Ziduo Liu

**Affiliations:** 1 State Key Laboratory of Agricultural Microbiology, College of Life Science and Technology, Huazhong Agricultural University, Wuhan, Hubei, P. R. China; 2 Key Laboratory of Protection and Utilization of Biological Resources in Tarim Basin of Xinjiang Production and Construction Corps, College of Life Science, Tarim University, Alar, Xinjiang, P. R. China; 3 Key Laboratory of Marine Biogenetic Resources, the Third Institute of Oceanography, State Oceanic Administration, Xiamen, Fujian, P. R. China; University of Minnesota, United States of America

## Abstract

Glyphosate, a broad spectrum herbicide widely used in agriculture all over the world, inhibits 5-enolpyruvylshikimate-3-phosphate synthase in the shikimate pathway, and glycine oxidase (GO) has been reported to be able to catalyze the oxidative deamination of various amines and cleave the C-N bond in glyphosate. Here, in an effort to improve the catalytic activity of the glycine oxidase that was cloned from a glyphosate-degrading marine strain of *Bacillus cereus* (BceGO), we used a bacteriophage T7 lysis-based method for high-throughput screening of oxidase activity and engineered the gene encoding BceGO by directed evolution. Six mutants exhibiting enhanced activity toward glyphosate were screened from two rounds of error-prone PCR combined with site directed mutagenesis, and the beneficial mutations of the six evolved variants were recombined by DNA shuffling. Four recombinants were generated and, when compared with the wild-type BceGO, the most active mutant B3S1 showed the highest activity, exhibiting a 160-fold increase in substrate affinity, a 326-fold enhancement in catalytic efficiency against glyphosate, with little difference between their pH and temperature stabilities. The role of these mutations was explored through structure modeling and molecular docking, revealing that the Arg^51^ mutation is near the active site and could be an important residue contributing to the stabilization of glyphosate binding, while the role of the remaining mutations is unclear. These results provide insight into the application of directed evolution in optimizing glycine oxidase function and have laid a foundation for the development of glyphosate-tolerant crops.

## Introduction

Glyphosate (N-phosphonomethylglycine) has been extensively applied worldwide as a broad-spectrum herbicide since 1974 [1]. Glyphosate inhibits 5-enolpyruvylshikimate-3-phosphate synthase (EPSPS) of the shikimate pathway [2], catalyzing the transfer of the enolpyruvyl moiety of phosphoenolpyruvate (PEP) to shikimate-3-phosphate (S3P). The mechanism by which glyphosate inhibits EPSPS in a reversible reaction indicates that glyphosate acts as a competitive inhibitor of phosphoenolpyruvate (PEP) to occupy the PEP binding site of EPSPS [3]. Currently, three glyphosate-resistant strategies have been used in transgenic crops: (i) the overproduction of EPSPS in plants such as a series of tolerant cell lines of *Nicotiana tabacum* [4], and *Petunia hybrida* [5,6], or a foreign EPSPS from bacteria with high glyphosate tolerance, for instance, through the expression of *Agrobacterium* sp. strain CP4 EPSPS [7-9] and a mutant of EPSPS from *Ochrobactrum anthropi* in transgenic plants [10]; (ii) N-acetylation of glyphosate by an evolved glyphosate N-acetyltransferase (GAT) from *Bacillus licheniformis*, conferring glyphosate-resistance to transgenic plants of *Arabidopsis*, tobacco, and maize by introducing the *gat* genes into them [11,12]; and (iii) expression of a glyphosate detoxification enzyme metabolized glyphosate in transgenic plants, such as the Monsanto's patent of glyphosate oxidoreductase (GOX) [13] and the evolved glycine oxidase from *Bacillus subtilis* (GO, EC 1.4.3.19) [14,15]. GOX and GO can both cleave the carbon-nitrogen bond in glyphosate and yield aminomethylphosphonic acid (AMPA) which is considered to be much less phytotoxic than glyphosate for most plant species [16]. Additionally, the mode of action of GOX and GO can endow with glyphosate-resistance to transgenic crops and predicted to reduce herbicide glyphosate residues [17].

Glycine oxidase (GO) is a FAD-dependent flavoprotein that catalyzes the oxidative deamination of glycine, short chain D-amino acids (e.g. D-alanine, D-proline, D-valine, etc.) and primary or secondary amines to yield the corresponding α-keto acid and hydrogen peroxide. GO, the first enzyme, plays a role in the biosynthesis of the thiazole ring of thiamine pyrophosphate [18]. The three dimensional structure of GO from *Bacillus subtilis* (BsuGO) was known and provided insights into its active sites as well as the mode of interaction with its substrates [18,19]. Despite showing a modest sequence similarity with sarcosine oxidase (MSOX, EC 1.5.3.1) [20], D-amino acid oxidase (DAAO, EC 1.4.3.3) and D-aspartate oxidase (DASPO, EC 1.4.3.1) [21], GO shares substrate specificity with these flavooxidases and seems to have a substrate preference for amines of a small size, such as sarcosine and glycine. Based on high resolution three-dimensional structures of BsuGO (PDB: 1RYI), Pollegioni et al. used rational design and site saturation mutagenesis to improve the properties of BsuGO to oxidize glyphosate by modulating the substrate preference exerted upon the entrance of the active site residues, and obtained the evolved variant G51S/A54R/H244A with a 175-fold decrease in *K*_m,app_ and a 210-fold increase in catalytic efficiency (*k*_cat_/*K*_m_) against glyphosate over the wild-type GO [14]. 

In the present study, we used glyphosate as a sole nitrogen source and isolated a glyphosate-degrading strain of *Bacillus cereus* HYC-7, and based on the report by Pollegioni et al. [14], cloned and characterized the wild-type BceGO, which, however, only showed a low oxidase activity on glyphosate. Then in the absence of accurate structural information of BceGO, we utilized directed evolution to engineer its substrate preference and activity on glyphosate. Molecular diversity was generated by two rounds of error-prone PCR random mutagenesis, and the beneficial mutations were combined and recombined with site-directed mutagenesis and DNA shuffling, together with a bacteriophage T7 lysis-based method for high-throughput screening of oxidase activity. Finally thirteen mutants with higher oxidase activity on glyphosate than the wild-type BceGO were obtained, and mutant B3S1 was found to possess the maximum activity, with a 160-fold increase in substrate affinity and a 326-fold enhancement in catalytic efficiency.

## Materials and Methods

### Chemicals, strains, plasmids and culture conditions

Glyphosate, glycine, sarcosine, D-alanine, o-dianisidine dihydrochloride, horseradish peroxidase and FAD were obtained from Sigma (U.S.A.). Strains, bacteriophage and plasmids used in this study are listed in Table 1. *Bacillus cereus* HYC-7 was isolated from the marine sediment sand supplied by the Marine Culture Collection of China, and has been deposited in China Center for Type Culture Collection (CCTCC AB 2013009). *E. coli* was cultured in Luria-Bertani medium at 37°C, and the bacteriophage T7 was grown as described previously [22]. 

**Table 1:** Bacterial strains, bacteriophage and plasmids used in the study.

Strains, bacteriophage and plasmids	Characteristics	Source
*Bacillus cereus* HYC-7	Glyphosate-degrading	Lab collection
*E.coli* DH5α	Host of gene cloning and mutant library	Lab collection
*E.coli* BL21 (DE3)	Host of protein expression	Lab collection
Bacteriophage T7	Linear double-stranded DNA	Lab collection
pGEX-6P-1	Expression vector, Amp^R^	Lab collection
pGEX-6P-BceGO	GO cloned from *Bacillus cereus*	This work
pGEX-6P-B3S1	Evolved GO from screened	This work

### Construction of the BceGO mutant libraries

#### (i): Random mutagenesis

The error-prone PCR was performed as previously described with some modifications [23]. The sequences of the two primers used in random mutagenesis were BceGO-F (5’-CGCGGATCCATGTGTAAGAAGTATGATGTAGCGAT-3’) and BceGO-R (5’-CCGCTCGAGCTAAACTCTCCTAGAAAGCAATGAAT-3’), the *Bam*HI and *Xho*I sites are in italic and underlined. The amplification mixture (100 µl) was composed of 20 nM primers, 0.2 mM dGTP and dCTP, 0.1 mM dATP and dTTP, 2 U *Taq* DNA polymerase and *Taq* buffer containing 5 mM MgCl_2_ and 0.5 mM MnCl_2_. The PCR procedure was performed in a thermal cycler (Bio-Rad Laboratories Inc.) for 30 cycles of (94°C for 30 sec, 57°C for 30 sec, and 72°C for 70 sec). PCR products were purified, digested with *Bam*HI and *Xho*I, cloned into pGEX-6P-1, and transformed into *E.coli* DH5α to create the random mutant library. In the first round of PCR-based random mutagenesis, pGEX-GO was used as the template, and a new mutant with improved catalytic efficiency against glyphosate was obtained by combining the beneficial mutation sites of variants, which was used as the starting-point for the second round of random mutagenesis.

#### (ii): Site-directed mutagenesis

Two of single-point BceGO variants (G51R and D60G) were combined by a rapid PCR-based site-directed mutagenesis [24] using mutant D60G as the template. The mutagenesis primers used were: G51R-F (5’- GCTGCTGGTTTACTTCGTGTTCAGGC-3’), and G51R-R (5’- ACGAAGTAAACCAGCAGCTGCTTTTG-3’), which were designed according to mutant G51R. The mutation positions are underlined, which were designed according to the mutant G51R. The two-point mutant was designated as B1R and validated by DNA sequencing.

#### (iii): DNA shuffling

DNA shuffling was performed following the procedure described by Stemmer with some modifications [25,26]. The variants with improved oxidase activities on glyphosate were selected from the second round of random mutant library, used as DNA shuffling templates and amplified with universal primers (6P-1F: 5’-ATCCTCCAAAATCGGATCTGGAA-3’ and 6P-1R: 5’-GGCAGATCGTCAGTCAGTCACG-3’). The purified PCR products were mixed equally, and then prepared for DNA fragmentation by ultrasonic treatment at 0°C for 40 min [27,28]. The fragments between 100~200 bp were purified using gel purification column (Axygen), and were reassembled by primerless PCR, which was performed in a thermal cycler (Bio-Rad Laboratories Inc.) as follows: 94°C for 3 min, 60 cycles of (30 sec 94°C, 30 sec 40°C, 20 sec + 1 sec per cycle 72°C), and 72°C for 10 min. After that, 5 µL of unpurified reassembly reaction mixture was used as the template to amplify the full-length sequence with primers BceGO-F/BceGO-R. The PCR amplification products were required to be observed at a single band of 1.1 kb, then were purified by DNA gel purification kit, digested by *Bam*HI and *Xho*I before being purified again. The purified products were ligated into the expression vector pGEX-6P-1, which was digested with *Bam*HI and *Xho*I sites, and then the resulting constructs were transformed into *E. coli* DH5α for screening.

### Screening of the evolved BceGO variants

A rapid and sensitive enzyme-coupled colorimetric assay was performed for high-throughput screening of evolved BceGO mutants toward glyphosate from the mutant library. The resulting library of BceGO mutants were expressed into 96 deep-well plates (containing 0.6 ml Luria-Bertani medium) and transferred onto Luria-Bertani agar plates as corresponding copies, followed by an overnight growth at 37°C. When the cultures grew to saturation, both IPTG (at a final concentration of 0.1 mM) and the bacteriophage T7 (above 100 particles per cell) [22] were added into 96 deep-well plates to synchronize the induction of recombinant mutants with the release of the lysis of the host *E.coli* DH5α at 37°C with shaking for 6 h. 

To screen for the improved mutants on glyphosate, the oxidase activity of BceGO mutants was accessed as follows: an aliquot of 159 µL of lysis cell extracts was transferred to the corresponding well of a microtiter plate, followed by the addition of 20 µL of 50 mM glyphosate (at a decreasing substrate concentration gradient in sequential rounds of screening), 20 µL of 0.32 mg/mL o-dianisidine dihydrochloride, and 1 µL of 5 unit/mL horseradish peroxidase in 50 mM disodum pyrophosphate buffer at pH 8.5, and an overnight incubation at 25°C. The absorbance change at 450 nm for each well in the microtiter plates was measured and compared with the control (harboring wild-type BceGO or containing the empty vector pGEX-6P-1) [14]. Mutants that outperformed the wild-type were selected for further activity analysis.

### Enzyme expression and purification

 The pGEX-6P-1 expression plasmids inserted into wild-type BceGO and variants were transferred to the *E.coli* BL21 (DE3). The recombinant strains were grown at 37 °C in Luria-Bertani medium containing 100 µg/mL ampicillin until the exponential phase, followed by the addition of IPTG to a final concentration of 0.1 mM, induction in 22 °C for 8 h, and then the collection of the cells by centrifugation. Then the cell pellets were suspended with 50 mM disodum pyrophosphate buffer at pH 8.5 and crude extracts were lysed with a high pressure homogenizer (NiroSoavi, Italy), followed by the addition of 5 µM FAD, 2 mM 2-mercaptoethanol, and 50 U deoxyribonuclease I into the supernatant of the lysate. Subsequently, the cellular debris was removed by centrifugation and the supernatant was incubated for 1 h at 4 °C with 1 mL GST∙Bind Resin. After the column materials were washed with 50 mM disodum pyrophosphate buffer at pH 7.5, the recombinant GO proteins were treated with 200 U PreScission protease at 4°C overnight. Finally, the purified GO and variants were eluted from the beads with 50 mM disodum pyrophosphate buffer at pH 8.5 and 10% glycerol. The purity of GOs was tested by SDS-PAGE and Coomassie brilliant blue staining. The concentration of GOs was determined with Bradford assay [29].

### Enzyme characterization and kinetic parameters

Wild-type BceGO and mutants activities were measured spectrophotometrically via determination of H_2_O_2_ produced by the BceGO reaction with an enzyme-coupled assay using horseradish peroxidase and o-dianisidine dihydrochloride [30]. One unit of GO corresponds to the amount of enzyme that converts 1 µmol of substrate (glycine or oxygen) or that produces 1 µmol of hydrogen peroxide per minute at 25°C. The specific activity of wild-type BceGO and mutants was assayed using four different substrates in 200 µL reactions mixtures in a 96-well microtitre plate, i.e. each well contained 20 µL 100 mM substrate solution, 20 µL 0.32 mg/ml o-dianisidine dihydrochloride, 1 µL 5 unit/ml horseradish peroxidase in disodum pyrophosphate buffer (50 mM, pH 8.5), and a fixed amount of enzyme, followed by the supplementation of 50 mM disodum pyrophosphate buffer at pH 8.5 to reach a final volume of 200 µL. After the mixture was incubated at 25°C for 60 min, the change in absorbance at 450 nm was recorded by using a Thermo Multiskan Spectrum plate reader. 

The kinetic parameters of wild-type BceGO and variants were measured using a fixed amount of enzyme and four substrates at a different concentrations (glycine, 0~300 mM; glyphosate, 0~600 mM; sarcosine, 0~300 mM; D-alanine, 0~600 mM ). Activity was assayed using the H_2_O_2_ produced in the GO reaction as reported [30]. The values of *V*_max_ and *K*_m_ were calculated using the program GraphPad Prism version 5.00 for Windows (GraphPad Software, San Diego, CA), and the *V*_max_ values were converted to *k*_cat_ values for normalization with respect to the kinetic parameters.

The effects of temperature and pH on the activities of the wild-type BceGO and variants were evaluated at different temperatures (0~70°C) and at different pH values by using 0.2 mM Na_2_HPO_4_-0.1 mM citrate buffer (pH 4.0~8.0), and 50 mM disodum pyrophosphate─NaOH buffer (pH 8.0~11.0). The thermal and pH stabilities GOs were assayed by incubating the enzyme at a temperatures range of (0~70°C) for 1 h, and with different pH buffers (pH 4.0~11.0) at 0 °C for 6 h, respectively, and then its residual activity and relative activity were determined and calculated.

### Molecular modeling and docking analysis

The homology module in MOE 2010.10 (Chemical Computing Group Inc., Montreal, Canada) was applied to build the 3D structure of mutant B3S1. The glycine oxidase structures from *Bacillus subtilis* (PDB code: 1RYI and 3IF9) [14,19] exhibited the highest identity (31%), and were thus considered to be the most appropriate templates. The docking, refinement of docked poses and the binding mode analysis of B3S1-glyphosate complex were performed with the docking and LigX module in MOE. The hydrogen bond pattern, solvent accessibility were assessed by analyzing the structural features of the B3S1 model, which analyses were carried out using ligand interaction analysis in MOE [31] and ASAView [32], respectively. The structural states were classified into three types based on the calculated solvent accessibility values (cut-off values, CV) of each residue, and the three-state model was created as previously described [33]: (i) the buried state (B), where the solvent accessibility value of each residue is 0≤CV≤9%; (ii) the intermediate state (I), where the value is 9%≤CV≤36%; and (iii) the exposed state (E), where the value is 36%≤CV≤100%.

## Results and Discussion

### Directed evolution and screening of evolved BceGO

A key process in directed evolution is the generation of genetic diversity using ‘irrational’ design approaches, such as random mutagenesis, DNA recombination, and a rapid and sensitive screening method so that the desired properties produced by residue substitutions can be detected [34]. In the present work, based on an insightful report about the application of rational design and site saturation mutagenesis in the evolution of BsuGO to obtain a variant with a high catalytic efficiency on glyphosate [14], we have altered BceGO’s substrate specificity towards glyphosate by using ‘irrational’ design methods focusing on sequential rounds of random mutagenesis and recombination. Firstly, we used a bacteriophage T7 lysis-based method for high-throughput spectrophotometric assay of oxidase activity, which facilitates the screening process and is able to handle massive clones. Secondly, we employed a directed evolution approach of sequential random mutagenesis and DNA shuffling to modulate the substrate speciﬁcity of BceGO towards the herbicide glyphosate. 

Error-prone PCR was used to create the first generation random mutant library of BceGO with an average of 1 to 2 amino acid substitutions per mutant for the majority of library as a whole. The resulting 14000 clones were screened for oxidase activity toward glyphosate at a screening concentration of 50 mM, with the absorbance of oxidized o-dianisidine dihydrochloride at 450 nm as an indicator of enzymatic oxidizability potential. The two active clones, 22D11 (G51R) and 23B1 (D60G), showed a redder colour than the wild-type BceGO, and then the two mutant sites were combined by site-directed mutagenesis and designated as B1R, which was used as the starting template for the second round of error-prone PCR. For evaluating the mutation frequency of the second round of random mutagenesis, inserts from 10 randomly picked clones were sequenced, resulting in 1~3 amino acid substitutions. Of approximately 16000 clones screened with the substrate glyphosate concentration at 10 mM, six mutants with a potential increase in oxidase activity were selected and identified by DNA sequencing before DNA shuffling (Table 2). 

**Table 2:** Amino acid substitutions of BceGO variants obtained by random mutagenesis, site directed mutagenesis, DNA shuffling and screening for enhanced specific activity on glyphosate.

Method	Mutant	Amino acid substitution
First round error-prone PCR	22D11	G51R
	23B1	D60G
Site directed mutagenesis	B1R	G51R, D60G
Second round error-prone PCR	B2R3	G51R, D60G, I111T, I284L, Q346R
	B2R6	G51R, D60G, D121G, S122P
	B2R11	G51R, D60G, K133R, V262I
	B2R14	G51R, D60G, T118A, K133R
	B2R23	G51R, G60S, I198V, E357G
	B2R81	G51R, G60S, S210P, M267T
Third round DNA shuffling	B3S1	G51R, G60S, T118A, K133R, I198V, V262I, I284L, L307S, E357G
	B3S4	G51R, G60S, D121G, S122P, H164Y, M267T
	B3S6	G51R, G60S, K133R, S210P, R250G, V262I
	B3S7	G51R, D60G, T118A, K133R, I284L

To recombine the beneficial mutations generated by error-prone PCR and enhance the enzyme’s activity against glyphosate, the coding genes of the six improved mutants were subjected to DNA shuffling generating a recombinant DNA library of about 20,000 clones, which were preliminarily screened for activity toward glyphosate at a 2 mM concentration in the procedure described above. After screening about 10000 clones in the microtiter plates, ten recombinants with improved activity were obtained, and after second screening with 0.5 mM glyphosate, four shuffled mutants (B3S1, B3S4, B3S6 and B3S7) were selected for purification (Table 2). Four recombinants contained 3.5 ± 0.5 crossovers on average at a range of 3~4 [35], in which, only three point mutations arose during shuffling, at a mutation frequency of 0.27%. For instance, the most improved variant B3S1 contained mutations of G51R/G60S from B2R23 or B2R81, K133R/V262I from B2R11, I284L from B2R3 and I198V/E357G from B2R23, and the substitution of L307S due to random point mutagenesis in the shuffling process. 

### Expression and characterization of wild-type BceGO and evolved enzymes

After the first round of error-prone PCR was performed, two mutants selected from these improved variants and wild-type BceGO were purified and characterized as described above, all of which exhibited increased catalytic efficiency on glyphosate (Table 3). When compared with wild-type BceGO, the two single point mutants of 22D11 (G51R) and 23B1 (D60G) that were screened from the first round of random mutant library showed a 5.27- and 5.61-fold increase in activity against glyphosate, respectively, while mutant B1R exhibited a 17-fold increase in activity on glyphosate, a 321-fold decrease in the catalytic efficiency toward glycine, and a 151-fold enhancement in the specificity constant (the *k*_cat_/*K*_m_ ratio between glyphosate and glycine, see last column in Table 3).

**Table 3:** The apparent kinetic parameters on glycine and glyphosate measured for wild-type BceGO and variants obtained by random mutagenesis, site saturation mutagenesis and DNA shuffling.

	Glycine		Glyphosate	
	*k*_cat,app_ (min^-1^)	*K*_m,app_ (mM)	*k*_cat,app_ (min^-1^)	*K*_m,app_ (mM)
Wild-type	8.17 ± 0.31	1.04 ± 0.17	5.72 ± 0.42	84.79 ± 4.25
22D11	1.16 ± 0.05	54.6 ± 3.47	2.95 ± 0.21	8.29 ± 0.27
23B1	4.56 ± 0.38	0.99 ± 0.04	7.15 ± 0.62	18.88 ± 2.52
B1R	0.44 ± 0.03	58.5 ± 5. 26	2.78 ± 0.46	2.45 ± 0.15
B2R11	1.86 ± 0.09	105.6 ± 7.31	3.83 ± 0.17	2.77 ± 0.21
B2R14	1.35 ± 0.12	92.5 ± 7. 43	4.16 ± 0.14	2.17 ± 0.37
B2R23	13.02 ± 0.96	101.8 ± 8.29	30.80 ± 1.33	3.80 ± 0.26
B2R81	5.41 ± 0.83	134.4 ± 10.33	7.27 ± 0.75	4.37 ± 0.30
B3S1	5.43 ± 0.79	41.55 ± 3.32	11.67 ± 0.98	0.53 ± 0.03
B3S4	5.68 ± 0.64	80.43 ± 5.01	12.99 ± 1.14	1.37 ± 0.07
B3S6	10.14 ± 1.32	138.1 ± 12.16	13.22 ± 1.78	1.69 ± 0.08
B3S7	2.30 ± 0.31	41.64 ± 2.10	4.63 ± 0.39	0.57 ± 0.02

**Figure 1:**
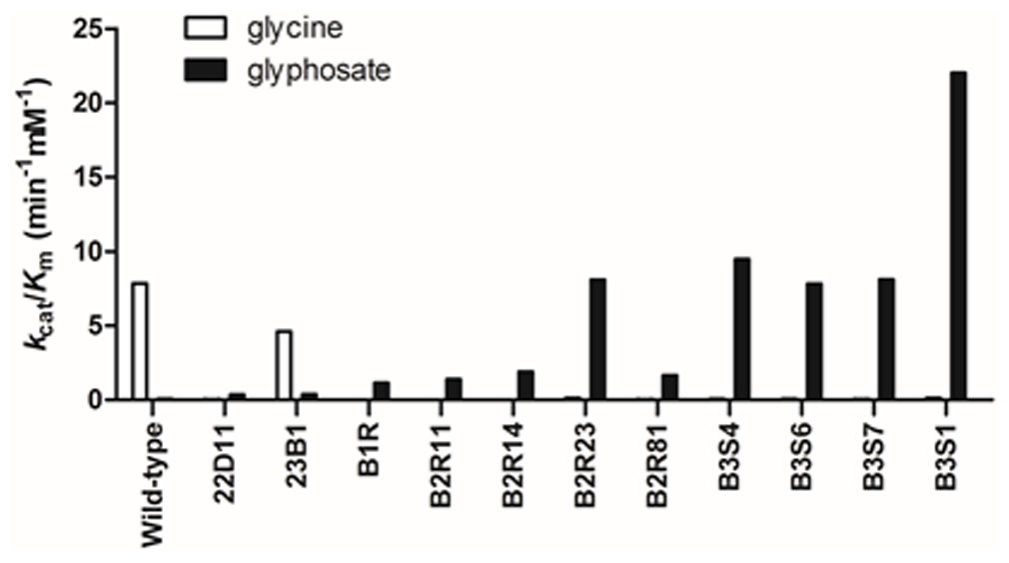
Comparison of catalytic efficiency (the ratios of *k*_cat_/*K*_m_) of wild-type BceGO and evolved variants.

Starting from mutant B1R, a second round of error-prone PCR was performed, and six mutants (B2R3, B2R6, B2R11, B2R14, B2R23 and B2R81) showed improved activity on glyphosate as compared with wild-type BceGO. For all the six variants, the changes of *k*_cat,app_ parameters were prominent for the substitution of G60S introduced in the second round of random mutagenesis, and the *k*_cat,app_ value of B2R23 was 11.08-fold that of the starting template (mutant B1R). These results demonstrated that BceGO had a considerable evolutionary landscape for improving kinetic efficiency on glyphosate. Further promotion of glyphosate oxidase activity by DNA shuffling was apparent in the third generation, obtaining four recombinants (B3S1, B3S4, B3S6 and B3S7) with a higher activity than those in the second generation. As shown in Table 3 and Figure 1, variation in the kinetic parameters of recombinants can reflect how the catalytic efficiency on glyphosate was improved: a significant decrease in *K*_m_ for all recombinants, and a high *k*_cat_ value for all mutants except B3S7. Especially, B3S1 demonstrated a 160-fold increase in substrate affinity for glyphosate, a 326-fold increase in catalytic efficiency towards glyphosate and a significant enhancement in the specificity constant over the wild-type BceGO, achieving the goal of efficient oxidation of glyphosate by evolution of glycine oxidase. 

To assay the substrate specificity of the wild-type BceGO and the most improved variant B3S1, the activities of BceGO and B3S1 were determined on four substrates at a fixed concentration(100 mM) using the spectrophotometric method coupled with horseradish peroxidase and o-dianisidine. BceGO presented a specific activity of 0.28 U mg^-1^ on glycine, which was less than BsuGO (0.8 U mg^-1^) [20] and the glycine oxidase from *Geobacillus kaustophilus* (GOXK) (11.85 U mg^-1^) [36]. But it showed a relatively higher activity on sarcosine (0.83 U mg^-1^) than other substrates, which was in line with a previous finding about BsuGO [20]. Among the tested substrates of glycine and glyphosate, variant B3S1 exhibited a 1-fold increase in specific activity on glyphosate, but a lower specific activity than the wild-type BceGO on glycine, sarcosine and D-alanine (Table 4). 

**Table 4:** Specific activities of wild-type BceGO and variant B3S1 toward four substrates.

	Specific activity (U/mg)^a^	
Substrate	BceGO	B3S1
Glycine	0.28 ± 0.05^b^	0.14 ± 0.02
Glyphosate	0.12 ± 0.02	0.24 ± 0.04
Sarcosine	0.83 ± 0.03	0.12 ± 0.02
D-alanine	0.22 ± 0.03	0.14 ± 0.02

^a^Enzyme assay was performed in 50 mM disodum pyrophosphate buffer at pH 8.5. One unit of specific activity was defined as the amount of enzyme converting 1 µmol of substrate per minute at 25°C.

^b^SDs are shown after the specific activities.

### Effects of temperature and pH on enzyme activity and stability

**Figure 2:**
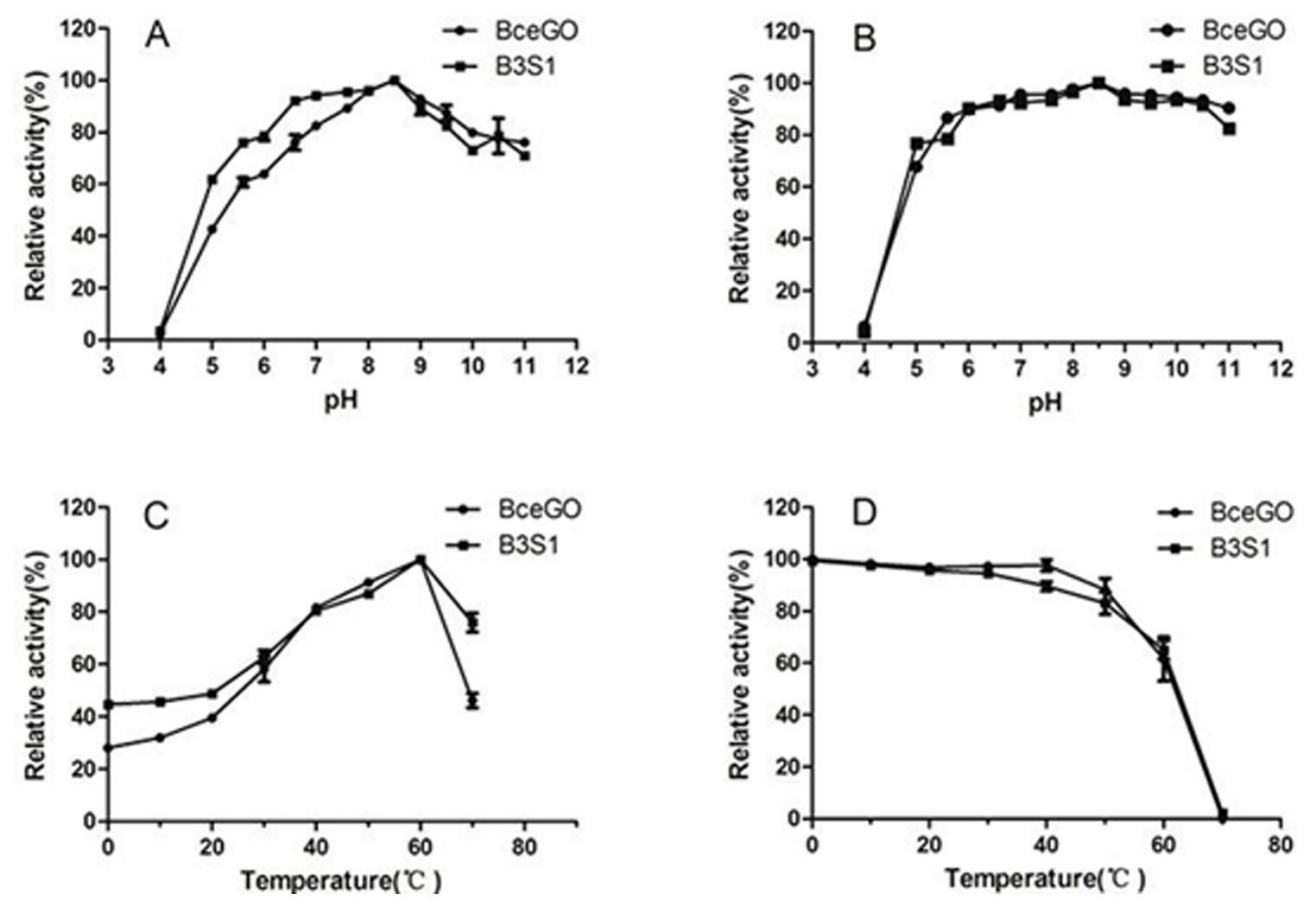
Effect of pH and temperature on activity and stability of wild-type BceGO and evolved variant B3S1. **A**. The optimal pH. Enzyme activity was determined with 100 mM glyphosate at 25 °C and within a pH gradient range of 4.0~11.0 with the following buffers: 0.2 mM Na_2_HPO_4_-0.1 mM citric acid buffer for pH 4.0~8.0, and 50 mM sodium pyrophosphate buffer for pH 8.0~11.0. The maximum activity observed was taken as 100%. **B**. The pH stability. Enzymes were incubated at 0 °C for 6 h over a pH buffer range of 4.0~11.0, then the enzyme activity was determined with 100 mM glyphosate at 25 °C and the optimal pH. The maximum activity observed was taken as 100%. **C**. The optimal temperature. The enzymes were added to the reaction mixture and the reaction was carried out at an indicated temperature from 0 to 70 °C. Then the enzyme activity was determined with 100 mM glyphosate at 25 °C and the optimal pH. The maximum activity observed was taken as 100%. **D**. The temperature stability. Enzymes were incubated for 1h at indicated temperature from 0 to 70 °C and then the enzyme activity was determined with 100 mM glyphosate at 25 °C and in the optimal pH. The activity without treatment was taken as 100%. Error bars represent the SD of the mean calculated for three replicates. *Solid* *dots* represent wild-type BceGO, *solid* *blocks* represent variant B3S1.

The effects of pH and temperature on the enzyme activity and stability were examined, and the results are shown in Figure 2. Wild-type BceGO and B3S1 both had the same optimal activity at pH 8.5, and also exhibited similar pH stability profiles after 6 h incubation at 0 °C (Figure 2A). The optimal pH of BceGO (pH 8.5) was identical with GOXK [36] and close to BsuGO (pH 8.0) [20]. Similar to BsuGO (pH 6.5~9.5) [19] and GOXK (pH 6.0~9.0) [36], the BceGO retained more than 80% of the original activity after 6 h incubation at 0 °C over a pH range from 6.5 to 11.0, but showed a significant decrease in activity below pH 5.0 (Figure 2B). Both wild-type BceGO and B3S1 exhibited the maximum activity at 60 °C (Figure 2C), and retained it up to 50 °C after 1 h incubation. They also showed a similar curve in thermal stability, with the activity decreasing sharply above 60 °C (Figure 2D), indicating that they had a lower thermal stability at the high temperature than GOXK from an extremophile microorganism *Geobacillus kaustophilus* [36]. 

### Structure modeling analysis of evolved variant B3S1

**Figure 3:**
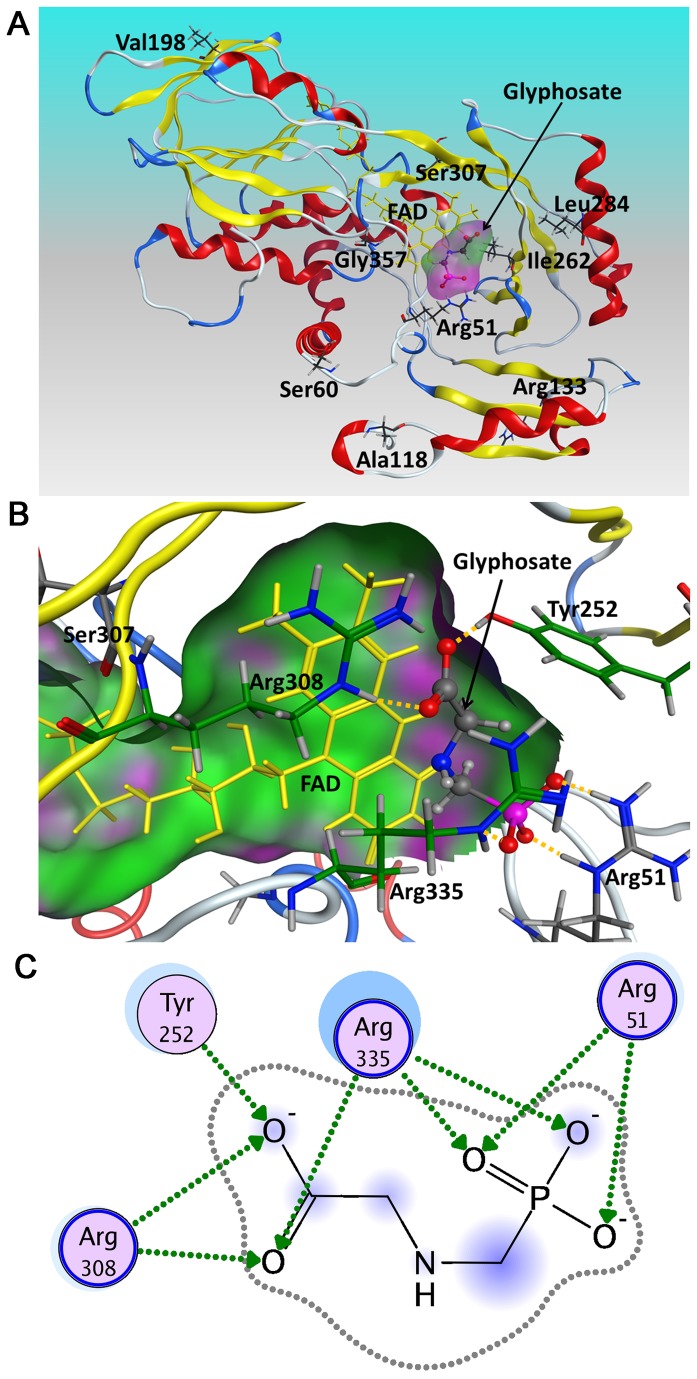
A. Docking analysis of glyphosate-B3S1 complex. The atoms of the nine amino acid mutations are shown with *stick* representation. The flavin cofactor is in *yellow* and the ligand glyphosate is shown with *ball-and-stick* representation. B. The model of variant B3S1 active site docking with the substrate glyphosate. The partial accessible space of the active site is shown in *green and purple*. The main active residues are shown with *stick* representation, and hydrogen bonds are represented in *yellow* *dotted* *lines*. C. 2D depiction of the glyphosate-residues interaction in variant B3S1. The schematic representation was generated using MOE and the residues are shown in *purple* *disks*. The hydrogen bonds are represented in *green* *dotted* *lines* with the *arrow* denoting the direction of the bond. The solvent-exposed surface of catalytic residues is drawn as a *halo-like* *disk* around the residue. The solvent exposure of ligand is expressed in *contour* *dotted* *line*, and the solvent exposure of substitution group is shown in *blue* *smudge*.

To identify the possible molecular basis for the enhancement of oxidase activity against glyphosate, we constructed a docking model of the B3S1-glyphosate complex based on the homology model (Figure 3A). Combined with the data of secondary structure predicted by the PSIPRED server [37], we found that two of the valuable mutations introduced into G51R/D60S were located on the loop connecting α2-α3 helix, and Arg^51^ was close to the active site, which established an electrostatic interaction and hydrogen bonds with the phosphonate group of glyphosate (Figure 3C). On the one hand, the guanidinium group of Arg^51^ contributed to the stabilization of glyphosate binding, which might enhance the affinity for glyphosate, but decrease the affinity for glycine. On the other hand, this polar residue was prone to provide partially positive charge to neutralize negative charge in the active site, thus increasing the cofactor’s redox potential [38]. The substitution of D60G was generated in evolved mutant 23B1 by replacing an acidic residue with a neutral residue without side chain, which contribute to the improved catalytic activity of GO, mainly because the loop connecting α2-α3 helix could possess a high mobility and bring a corresponding slight conformation change in the proximity of active site [14]. The mutations (G51R/D60G) close to the active site improved the catalytic efficiency of BceGO on glyphosate mainly by decreasing the *K*_m_ value (up to 35-fold in mutant B1R). 

In the second random mutagenesis, the replacement of G60S with a short polar side chain residue resulted in a corresponding increase in *k*_cat_ (mutants B2R23 and B2R81 in Table 3), which could be assumed that the new substitution of G60S can optimize the conformation at the active site entrance, thus improving the catalysis of BceGO on glyphosate. Besides, the mutations of T118A, K133R, I198V, V262I, I284L, V262I and E357G were far away from the active site and introduced in the second error-prone PCR, which may cause a slight conformational change and contribute to the promotion of catalytic activity on glyphosate.. While mutation of L307S adjoined the catalytic residue Arg^308^ (the corresponding residue is Arg^302^ in BsuGO), they were both located in a conservative loop WAGLRP, and they might be involved in the interaction with the substrate and formed a lid to cover the substrate binding site according to prior studies [18,19].

To analyze the location and accessibility of the mutations identified, and clarify the topological distribution of these mutated residues in variant B3S1, the relative solvent accessibility scores were predicted by ASAView [32]. The results showed that the three residues of Arg^133^, Val^198^, and Gly^357^ were located on the surface with a clearly higher calculated solvent accessibility value (75.9%, 64.4% and 47% respectively), and the four residues of Ala^118^, Ile^262^, Leu^284^, and Ser^307^ were in the buried state in the enzyme with a lower solvent accessibility value (from 0 to 4.8%). Only Arg^51^ and Ser^60^ were in the intermediate state in B3S1 structure whose the predicted solvent accessibility value was 12.7% and 29%, respectively, suggesting that from the structural point of view, except that Arg^51^ and Ser^60^ were in the vicinity of the entrance to the active site, and the other mutations were far away from the active centre, but these mutations also play a role in improving catalytic activity. Comparison of wild-type BceGO with B3S1 and other recombinants such as B3S4, B3S6 and B3S7 revealed some different kinetic properties toward glyphosate, i.e., mutations both close to and far away from the active site can effectively improve catalytic activity (Table 3). Our results have confirmed the observation that the substrate speciﬁcity of an enzyme can be modulated by a few mutations of residues [39,40]. Although random mutagenesis is targeting the entire coding sequence of the enzyme, only a few mutated residues form the substrate binding site, most mutated residues lie far away from active site [40]. Can these distant mutations improve the catalytic efficiency? The answer is that they might not only cause a subtle disruption in the spatial configuration of the active site, but also some fine alterations in the protein backbone and side chain, which can produce an effect on the protein secondary structure, cause subtle changes in the arrangement of the protein tertiary structure or the shape of the binding pocket, and finally lead to dramatic changes in the catalytic power of enzyme [41]. Therefore, based on the structural model, it could be deduced that while the mutations close to the active site appeared to be more useful in altering an enzyme’s substrate selectivity and catalytic activity, these distant mutations could also play an auxiliary role in improving or modifying the catalytic properties of the enzyme.

The schematic 2D representation of B3S1-glyphosate complex is shown in Figure 3C. As can be seen from the 2D depiction of B3S1-glyphosate complex, the side chain of Arg^308^ forms two H-bonds with the carboxylic group of glyphosate and this residue plays a primary role in substrate binding for enzymatic activity [14]. In addition to Arg^308^, the carboxylic group of glyphosate might also form H-bonds with Tyr^252^ and Arg^335^ side chains. The arginine introduced at position 51 (instead of glycine) is at a suitable location to interact with the phosphonate group of glyphosate, with the corresponding substitution of G51R in BsuGO close to the active site entrance in GO [14]. The guanidinium group of Arg^335^ also contributes to the stabilization of the phosphonate group and the carboxylate group of glyphosate (Figure 3B), and possesses the largest solvent-accessible surface areas (Figure 3C). 

## Conclusions

Here, in the absence of detailed structure information of BceGO, we conducted a rapid and sensitive screening for improved variants activity against glyphosate using a directed evolution approach of sequential random mutagenesis, site-directed mutagenesis and DNA shuffling, together with a bacteriophage T7 lysis-based method for high-throughput spectrophotometric assay coupled with horseradish peroxidase/*o*-dianisidine. A total of thirteen evolved variants were isolated from the mutant libraries and, when compared with wild-type BceGO, the most active mutant B3S1 possessed a 160-fold higher substrate affinity for glyphosate, a 326-fold higher catalytic efficiency against glyphosate and a 6,017-fold increase in the specificity constant (the *k*_cat_/*K*_m_ ratio between glyphosate and glycine), indicating that the BceGO substrate speciﬁcity and catalytic activity have been successfully engineered by using sequential evolution and selection to exploit the sequence space. The *k*_cat_/*K*_m_ value of G51S/A54R/H244A BsuGO variants for glyphosate is ^≈^ 120 mM•min^-1^ [14], which was expressed in *Medicago sativa* and acquired resistance to glyphosate [15]. Therefore, we basically achieved the goal for engineering the substrate specificity of BceGO toward the degradation of the herbicide glyphosate. Although BceGO and glyphosate oxidoreductase (GOX) are different in catalytic mechanism of glyphosate oxidation [13,42], the two enzymes share some similarities including (i) breakage of the C-N bond in glyphosate to generate the same product (AMPA and glyoxylate), (ii) the property of FAD-containing flavoenzymes, and (iii) a low sequence identity (20%) with BceGO and GOX. The evolved B3S1 we reported here shows a 5-fold lower *K*_m_ value for glyphosate than GOX (0.53 *versus* 2.6 mM, respectively), but compared with the diffusion-limited maximal value (10^9^ M^-1^ s^-1^), the *k*_cat_/*K*_m_ value of variant B3S1 for glyphosate is ^≈^ 22 mM•min^-1^, which is still of a great potential for further optimization by directed evolution.
